# Role of pathogenic oral flora in postoperative pneumonia following brain surgery

**DOI:** 10.1186/1471-2334-9-104

**Published:** 2009-06-29

**Authors:** Kinga Bágyi, Angela Haczku, Ildikó Márton, Judit Szabó, Attila Gáspár, Melinda Andrási, Imre Varga, Judit Tóth, Almos Klekner

**Affiliations:** 1 Faculty of Dentistry, Medical and Health Science Center, University of Debrecen, Debrecen, Hungary; 2 Pulmonary, Allergy and Critical Care Division, Department of Medicine, University of Pennsylvania, Philadelphia, PA, USA; 3 Institute of Medical Microbiology, Medical and Health Science Center, University of Debrecen, Debrecen, Hungary; 4 Institute of Inorganic and Analytical Chemistry, University of Debrecen, Debrecen, Hungary; 5 Department of Pulmonology, Medical and Health Science Center, University of Debrecen, Debrecen, Hungary; 6 Department of Oncology, Medical and Health Science Center, University of Debrecen, Debrecen, Hungary; 7 Department of Neurosurgery, Medical and Health Science Center, University of Debrecen, Debrecen, Hungary

## Abstract

**Background:**

Post-operative pulmonary infection often appears to result from aspiration of pathogens colonizing the oral cavity. It was hypothesized that impaired periodontal status and pathogenic oral bacteria significantly contribute to development of aspiration pneumonia following neurosurgical operations. Further, the prophylactic effects of a single dose preoperative cefazolin on the oral bacteria were investigated.

**Methods:**

A matched cohort of 18 patients without postoperative lung complications was compared to 5 patients who developed pneumonia within 48 hours after brain surgery. Patients waiting for elective operation of a single brain tumor underwent dental examination and saliva collection before surgery. Bacteria from saliva cultures were isolated and periodontal disease was scored according to type and severity. Patients received 15 mg/kg cefazolin intravenously at the beginning of surgery. Serum, saliva and bronchial secretion were collected promptly after the operation. The minimal inhibitory concentrations of cefazolin regarding the isolated bacteria were determined. The actual antibiotic concentrations in serum, saliva and bronchial secretion were measured by capillary electrophoresis upon completion of surgery. Bacteria were isolated again from the sputum of postoperative pneumonia patients.

**Results:**

The number and severity of coexisting periodontal diseases were significantly greater in patients with postoperative pneumonia in comparison to the control group (p = 0.031 and p = 0.002, respectively). The relative risk of developing postoperative pneumonia in high periodontal score patients was 3.5 greater than in patients who had low periodontal score (p < 0.0001). Cefazolin concentration in saliva and bronchial secretion remained below detectable levels in every patient.

**Conclusion:**

Presence of multiple periodontal diseases and pathogenic bacteria in the saliva are important predisposing factors of postoperative aspiration pneumonia in patients after brain surgery. The low penetration rate of cefazolin into the saliva indicates that its prophylactic administration may not be sufficient to prevent postoperative aspiration pneumonia. Our study suggests that dental examination may be warranted in order to identify patients at high risk of developing postoperative respiratory infections.

## Background

Patients requiring a prolonged surgical procedure with mechanical respiratory support frequently develop post operative or ventilator-associated pneumonia that increases morbidity, mortality and length of recovery [1-6]. While several different routes have been suggested, in most cases the pulmonary infection appears to result from aspiration of pathogens colonizing the oral cavity. However, there is still some discussion about the definitive evidence of a causal relationship between oral pathogens and post-operative pneumonia [7,8]. The human saliva harbors abundant bacterial flora [6,9,10] and aspiration of saliva containing pathogenic bacteria can lead to pneumonia [11-14].

Aspiration pneumonia-associated mortality is one of the most serious problems in elderly patients [15,16]. Hospital inpatients that undergo endotracheal intubation for long surgical procedures are also at increased risk of aspiration [17,18]. In neurosurgical patients, aspiration of oropharyngeal secretions can be facilitated by leakage around the endotracheal tube cuff, weakness of pharyngeal and coughing reflexes, impaired level of consciousness, and postoperative immobilization [7].

Poor oral hygiene and periodontal disease can increase the proliferation of pathogenic bacteria many of which can cause aspiration pneumonia. Indeed Inglis et al. [19,20] reported that the major factor in the development of pneumonia was not the type but the amount of bacteria aspirated. An increase in the number of oral pathogenic species has been demonstrated in elderly persons requiring nursing care [15,16]. Furthermore, professional oral care has been shown to reduce this number [21] and oral care decreased frequency of fever [22,23] and mortality rate from pneumonia in the elderly [23-25].

The therapeutic benefits of the first and second generation cephalosporins have been thoroughly investigated and their wide spectrum of efficacy distinguishes them from other antibiotics for perioperative prophylaxis [26-29]. Administration of cephalosporins for surgical procedures is a common practice for the prevention of infections, but the chosen antibiotic varies. We have previously found that after a single intravenous dose, a number of cephalosporins failed to accumulate in the saliva and sputum of patients at high concentrations, indicating insufficient local antibacterial coverage [30,31].

Here we investigated the cefazolin levels in serum, saliva and bronchial secretions postoperatively and compared them with the MIC of a range of bacteria isolated from the oral cavity of the patients. We also investigated whether a correlation exists between bacterial sensitivity to cefazolin and development of post-operative pneumonia. We hypothesized that presence and severity of periodontal disease increases the risk of oral pathogen aspiration during brain surgery in this population. In this study we aimed to clarify the importance of assessing periodontal status before neurosurgical procedures on older patients in order to identify those at high risk for developing postoperative pneumonia.

## Methods

### Patient recruitment

All procedures were approved by the Ethical Committee of the University of Debrecen, Hungary and every patient signed an Informed Consent Form prior to recruitment to the study. Patients recruited into the study were non-smokers, aged older than 60 years and were diagnosed with the presence of an intracranial solitary extraaxial supratentorial tumor, Glasgow Coma Scale (GCS) 15; scheduled for elective routine craniotomy and tumor removal between January 2006 and August 2007 on the Neurosurgical Department of the University of Debrecen. Morbidly obese patients, smokers and who had diabetes mellitus, alcohol abuse, any immune deficiency, preexisting pulmonary conditions (e.g. chronic obstructive pulmonary disease, COPD), heart insufficiency and in whom prompt postoperative extubation could not be anticipated or neurological complications appeared, were excluded from the study. The recruited patients had normal preoperative chest X-ray and their laboratory parameters were in the physiological range. Patients had no lower cranial nerve palsy, so they had normal gag and coughing reflexes. Only those patients were recruited finally in the study that had no changes in the neurological status after the operation. The perioperative feeding status of each recruited patient remained normal as well. All patients were observed and treated in the Intensive Care Unit of the Neurosurgical Department after surgery.

**Figure 1:**
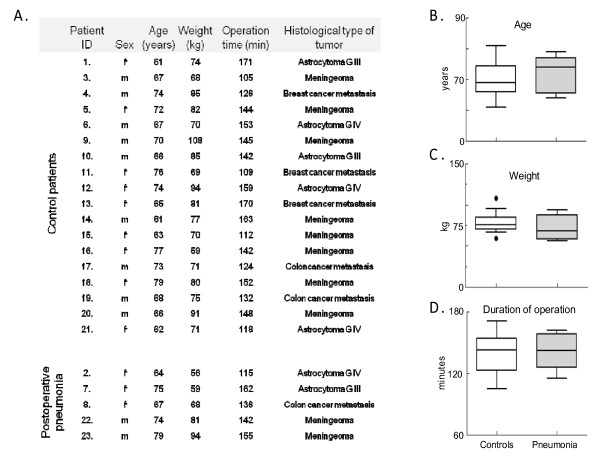
**Clinical parameters of patients**. Patients that developed pneumonia had comparable age, weight and duration of operation to controls that had no postoperative pneumonia. (A): Patients demographic data in a table format. (B-C): The patients who developed postoperative pneumonia were matched according to sex, age, weight and length of brain surgery. Data were compared using the non-parametric Mann-Whitney test and expressed as "box and whiskers: 10–90 percentile".

Following brain surgery a cohort of eighteen sex- (Figure 1A), age- (Figure 1B), tumor type (Figure 1A), weight- (Figure 1C) and operation-time (Figure 1D) matched patients without postoperative lung complications (control group) were compared to five patients having postoperative aspiration pneumonia developed within 48 hours after surgery (pneumonia group) (Figure 1). Pneumonia was diagnosed based on the guidelines of the American Thoracic Society by X-ray (new or progressive radiographic infiltrate), new onset of fever (body temperature = 38.0°C), symptoms of coughing with purulent sputum, chest pain and leukocytosis (white blood cell count > 10.0 G/L) [32]. Sputum samples were collected from pneumonia patients for bacterial culture by a pulmonologist. Patients diagnosed with pneumonia were treated promptly with ceftriaxone 80 mg/kg/24 hour followed by treatment with the appropriate antibiotic according to the results of the antibiotic susceptibility tests obtained from the bacterial cultures.

### Dental examination

Preoperative dental examinations were performed by the same experienced dentist on each patient awaiting neurosurgical procedures. In order to be able to quantify the severity of the periodontal disease in patient populations, a numeric scoring system based on commonly accepted disease severity definitions for evaluation of periodontal diseases was established. Periodontal conditions of the patients were categorized in five main diagnoses that were each given a numeric score. The following five conditions were diagnosed in the patients: *Dental calculus*: score „3” (presence of visible calcified deposit around the teeth); *Chronic gingivitis*: score „4” (redness and swelling around the gum without loss of connective tissue attachment). *Mild periodontitis*: score „5” (periodontal site with 3 millimeters of attachment loss and 4 millimeters of pocket depth). *Moderate periodontitis*: score „6” (teeth with interproximal attachment loss of 4 millimeters OR teeth with 5 millimeters of pocket depth at interproximal sites). *Severe periodontitis*: score „7” (teeth with interproximal attachment loss of 6 millimeters or more AND at least one tooth with 5 millimeters or more of pocket depth at interproximal sites). The "Disease Score" was then calculated as the sum of the scores of co-existing periodontal diseases in a patient (possible range: 0–25). In addition, patients received a "Severity Score", which equaled the number of their co-existing periodontal diseases (possible range: 0–5). For example a patient with dental calculus and mild periodontitis would have a Disease score of 8 and a Severity score of 2.

During dental examination saliva samples were collected for bacterial cultures. Bacterial strains were isolated preoperatively from the saliva of the patients and the minimal inhibitory concentrations (MIC) of cefazolin for each of the saliva-derived bacterial strains were determined.

### Determination of MIC

The MIC of cefazolin was determined with a standard broth microdilution method according to the guidelines of Clinical and Laboratory Standards Institute (CLSI) [33]. Cefazolin was diluted in sterile distilled water [34]. The interpretation criteria were in accordance with those suggested by the CLSI.

### Drug administration and postoperative sample collection

A single dose of cefazolin (15 mg/kg) was given intravenously to patients just prior to skin incision. Following the surgical procedures to safely reverse the muscle relaxant (*rocuronium*), patients received 0.015 mg/kg *neostigmine *and 0.0075 mg/kg *atropine*. Bronchial secretions were also aspirated from the trachea with a sterile suction-pipe through the endotracheal tube. Every patient recovered without severe neurological symptoms and GCS above 13 and was extubated promptly after the operation. During extubation, serum samples were obtained and saliva samples were collected from the pharynx above the cuff of the endotracheal tube.

The concentrations of cefazolin in the serum, saliva and bronchial secretion were determined and the levels of cefazolin were compared to the MIC values of the bacteria isolated preoperatively from the saliva. Bacteria were isolated again from the sputum of the five patients in whom postoperative pneumonia developed 48 hours after brain surgery. Sputum samples were carefully collected before ceftriaxone administration by a pulmonologist with a sterile suction-tube to avoid oral contamination. The isolated bacteria were compared to the bacteria isolated before the operation from the saliva of the same patients.

### Capillary electrophoresis

Serum, saliva, and bronchial secretion samples were stored at -20°C until analysis was performed. The concentration of cefazolin was determined by capillary electrophoresis as described previously [35]. Electrophoretic runs were performed no later than 4 hours after sample preparation. The optimal separation conditions for the determination of different cephalosporin concentrations using capillary electrophoresis were the subject of our earlier study [36,37]. The capillary electrophoresis instrument used in the current study was a CE3D model (Agilent, Waldbronn, Germany).

### Statistical analysis

Data from a cohort of control (n = 18) and pneumonia (n = 5) patients were compared using the non-parametric Mann-Whitney test. Unless otherwise stated, data were expressed as median and interquartile range. Correlations were made using regression analysis. Odds ratios were calculated using contingency table and the Chi-square test; one-sided p value was calculated. A p value of < 0.05 was accepted as significant.

## Results

Patients that developed pneumonia had comparable age, weight and duration of operation to controls. The final patient selection included 23 patients. The average age was 70.4 ± 5.9 years (mean ± SD), the average weight was 76.9 ± 12.5 kg (mean ± SD). The duration of the brain surgery ranged between 105 and 171 minutes of the recruited patients, with an average of 140.2 ± 19.7 minutes (mean ± SD).

Five patients who developed postoperative aspiration pneumonia (pneumonia group) were compared to eighteen patients without postoperative lung complications (control group) for their preoperative periodontal status, oral bacterial profile, cefazoline MIC value of the isolated bacteria and cefazolin concentration in the serum, saliva and bronchial secretion at time of extubation after surgery. The two patient groups we studied were carefully matched for sex, age, weight, histological diagnosis of the brain tumor and duration of operation (Figure 1).

**Figure 2:**
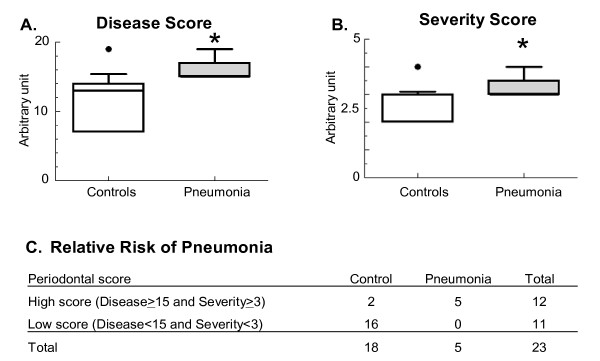
**Relative risk for postoperative pneumonia in relation to periodontal diseases**. Presence of periodontal diseases and disease severity were significantly greater in patients with postoperative pneumonia. (A-B): Data were expressed as "box and whiskers: 10–90 percentile" and compared using the non-parametric Mann-Whitney test (A): Disease score: periodontal diseases in every patient were evaluated. A score number was ordered to each diagnose, and the sum of the scores appears as "disease score". * = p = 0.0018 (B): Severity score is the number of co-existing periodontal diseases in each patient. * = p = 0.031; (C): Analysis of Relative Risk: "High score" patients had a Disease Score of ≥ 15 and a Severity Score of ≥ 3. Number of patients is shown in each groups. Chi-square test was performed, one-sided p value was calculated. *p < 0.0001; Relative risk: 3.5; confidence interval (95%): 1.085 to 11.29.

Presence of periodontal disease and disease severity were significantly greater in patients with postoperative pneumonia. Currently used methods to assess periodontal status may not accurately reflect a risk for aspiration pneumonia. We established five different main periodontal diagnoses in patients waiting for neurosurgery, and scores were assigned to each diagnosis. Both the Disease Score (Figure 2A) and the Severity score (Figure 2B) was significantly elevated in patients who developed postoperative aspiration pneumonia (p = 0.0018 and p = 0.031, respectively).

To calculate the risk of pneumonia, we divided the patients into a "High periodontal score" (Disease Score ≥ 15, Severity score ≥ 3) and a "Low periodontal score" group (Disease Score < 15, Severity score < 3). Analysis of the relative risk revealed that the "High periodontal score" patients had a significantly greater chance to develop pneumonia than the "Low periodontal score" patients (p < 0.0001, relative risk: 3.5 (confidence interval [95%]: 1.085 to 11.29) (Figure 2C).

**Figure 3:**
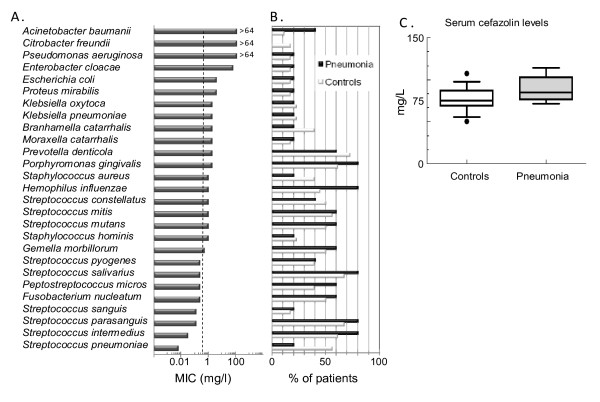
**Minimal inhibitory concentrations (MIC) of cefazolin and its concentration in serum**. Neither cefazolin sensitivity of the bacteria isolated from the saliva/bronchial secretions nor serum cefazolin levels were different between control and pneumonia patients. Bacteria were isolated from the saliva pre- and postoperatively. Bronchial secretion and serum were obtained post-operatively for bacterial culture and determination of cefazolin levels. (A): Distribution of isolated bacteria according to their MIC values. The antibiotic level of all the saliva and bronchial secretion samples fell below 0.5 mg/L; marked by the vertical dashed line. (B): Distribution of isolated bacteria according to percentage of patients with that particular species in each group. (C): Serum cefazolin levels are expressed as "box and whiskers: 10–90 percentile" and compared using the non-parametric Mann-Whitney test between control and pneumonia patients (n = 18 and 5, respectively).

Cefazolin levels were measured by capillary electrophoresis. Figure 3A shows the distribution of isolated bacteria according to their MIC values. While Gram positive bacteria predominated in most of the patients, Gram negative bacteria were also isolated from the oral cavity, several of which showed resistance for cefazolin (Figure 3B).

Presence of these "high MIC" bacteria was found in both the control and pneumonia groups and there was no relationship between the maximal MIC needed for bacterial coverage and development of postoperative pneumonia in the individual patients. High serum cefazolin levels (Figure 3C) did not correspond to cefazolin measured in the saliva. In fact the antibiotic levels in all the saliva samples were below the detection level, 0.5 mg/L (marked by a vertical dashed line in Figure 3A) indicating a low penetration of this antibiotic into the saliva. When serum cefazolin concentrations were compared between control and pneumonia patients (Figure 3C) we found no significant difference suggesting that there was no relationship between circulating cefazolin levels and presence of pneumonia after brain surgery.

**Figure 4:**
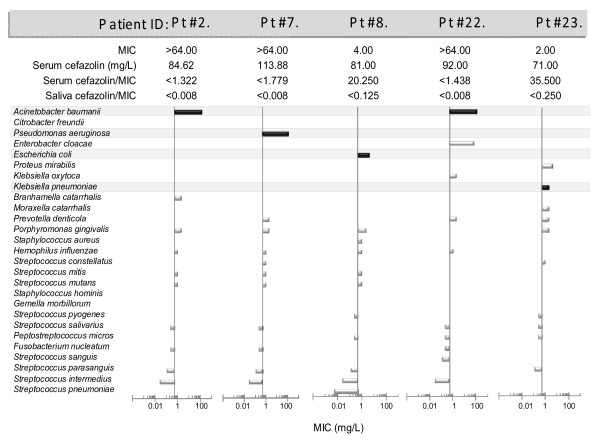
**Comparison of the minimal inhibitory concentration (MIC) of cefazolin regarding the bacteria isolated preoperatively from the saliva and postoperatively from the sputum**. Cefazolin resistant pathogenic, Gram negative bacteria isolated from the saliva pre-operatively, were also grown from the sputum of the postoperative pneumonia patients. The vertical axis of the graphs crosses the x axis at 0.5 mg/L. The antibiotic level of all the sputum samples fell below 0.5 mg/ml. The bars indicate the MIC value of bacteria obtained pre-operative from the saliva. Black bars denote the bacteria that were cultured from both the sputum and the saliva of the same patient.

The same cefazolin resistant Gram negative bacteria were isolated from the saliva pre-operatively and the sputum of the patients who developed postoperative pneumonia. In order to determine whether adequate serum cefazolin concentrations were achieved against the pathogenic bacteria in the pneumonia patients, we calculated the serum cefazolin/MIC ratio (using the MIC that was determined for the bacteria isolated from the sputum) in each of the patients. To provide adequate antibacterial protection, this value should ideally be greater than 1. Although circulating cefazolin/MIC ratios appeared adequate, the same ratios for the saliva of the patients were significantly below 1 (Figure 4D). In addition, comparison of the serum cefazolin/MIC ratio between control and pneumonia patients showed no difference (not shown).

## Discussion

In this study we investigated the significance of periodontal diseases in postoperative pneumonia and the effects of preoperative cefazolin prophylaxis on the oral pathogen flora in older patients after neurosurgical operation. Our results showed that the same pathogenic Gram negative bacteria isolated from the sputum of pneumonia patients were also isolated pre-operatively from the saliva suggesting aspiration etiology. There was no association between the type of bacteria isolated, their cefazolin sensitivity or the cefazolin serum levels and development of postoperative pneumonia. However, the severity of periodontal disease of the patients appeared a significant risk factor for postoperative pneumonia. Our study emphasizes the importance of preoperative oral health assessment and identification of patients at risk as this type of pneumonia can be prevented by proper care.

Bacterial bronchopneumonia is one of the most common infectious diseases in adults, with a well-known high morbidity and mortality, especially in old patients [38-40]. In a large-scale European study pneumonia accounted for approximately 50% of all intensive care unit (ICU) infections which significantly increased ICU death [41]. Although multiple risk factors are identified, the single most important one has been considered to be intubation [42]. Pneumonia also often appears as a postoperative complication after various surgical procedures [43,44]. Pneumonia can develop in surgical patients without previous lung disease or brain damage [18], but risk for aspiration is higher in older patients [17]. Further, age, sex, obesity, preoperative hospital stay, smoking, chronic obstructive pulmonary disease, other preexisting pulmonary conditions, type and duration of surgery and anesthesia, and nasogastric tube placement would each be considered a significant risk factor [44]. While aspiration of oral bacteria is one of the main etiologic factors in developing pneumonia in hospitals [3,45] the significance of existing periodontal disease is not completely clarified. In this study we selected elderly patients, >60 years of age, since periodontal diseases are known to be very common in this age group and there are often pathogenic bacteria identified in their saliva. Furthermore, after identification of the pneumonia patients, age, sex, weight, duration and type of operation were matched with the control group for comparison of their periodontal status.

Patients, who underwent cranial operations, were selected for investigation. As opposed to abdominal surgery, brain surgery is considered "clean", without open exposure to enteral pathogens [46]. Therefore this surgical population is particularly suitable to investigate the importance of enteral bacteria from the oral cavity and upper airways, in development of postoperative pneumonia. During long surgical procedures, saliva and oropharyngeal secretions accumulate in the pharynx above the cuff of the tube used for intratracheal ventilation. These secretions may then be aspirated because function of the lower cranial nerves can be impaired and so pharyngeal and coughing reflexes return slowly in this patient population. Additionally, the low level of consciousness and prolonged immobilization period after cranial surgery are all important factors in aspiration of saliva and oropharyngeal secretions and can increase the likelihood of developing pulmonary complications [47,48]. Thus, verification of the importance of aspiration etiology and the ability to identify patients who are at increased risk to develop postoperative pneumonia has a particularly high clinical importance in neurosurgical patients.

Although several methods for evaluation of oral hygiene were reported [49,50] problems in reliability and validity have also been noted [51]. The currently used methods to assess periodontal status range from simple visual evaluation of presence or absence of tongue coating [52] through manual probing [53] to elaborate bacterial cultures or sophisticated molecular biology techniques [54]. In our study a quantitative scoring assessment was established using generally accepted visual diagnosis and manual probing [55]. Presence of *Dental calculus, Chronic gingivitis, Mild periodontitis*, *Moderate periodontitis *and *Severe periodontitis *were diagnosed. The "Disease Score" was then calculated as the sum of the scores of co-existing conditions. In addition, patients received a Severity Score showing the number of their co-existing conditions. Based on these scores we divided the patients into a "High periodontal score" and a "Low periodontal score" group. Our results showed that the "High periodontal risk" patients had a significantly greater chance to develop pneumonia than the "Low periodontal risk" patients indicating that presence of severe periodontal diseases predisposed to postoperative pneumonia in our patient population. Furthermore, our periodontal scoring system appeared to have a good predictive value based on the calculated *sensitivity *(0.8889; CI [95%]: 0.6529 to 0.9862), *specificity *(1.000; CI [95%]: 0.4782 to 1.000), *positive predictive value *(1.000; CI [95%]: 0.7941 to 1.000) and *negative predictive value *(0.7143; CI [95%]: 0.2904 to 0.9633). Regarding the low n however, further validation of our scoring system is needed in a larger clinical trial. Nevertheless, these results confirmed that evaluation of periodontal status using a relatively simple visual scoring system may identify patients with high risk for developing postoperative pneumonia. Indeed, Gram negative bacteria isolated from the saliva of patients with high periodontal score before surgery, were also grown from the sputum after they developed pneumonia post-operatively, further suggesting that the infectious organisms originated from the oropharyngeal region.

These results also indicated that infection occurred in spite of cefazolin pretreatment in the pneumonia patients. The antibiotics most appropriate for prophylaxis of postoperative infections depend on the nature of the operation [56,57]. In aseptic (clean) operations, such as brain surgery, Gram-positive postoperative infections are the primary concern, and cefazolin has been recommended because of its excellent pharmacokinetics and good activity against Gram-positive pathogens, including staphylococci [46]. Although Gram negative bacteria are in general resistant to cefazolin it can be used also against some of them [30,47,57,58]. Single-injection prophylaxis given at the time of induction of anesthesia is effective, inexpensive, has relatively few side effects and does not induce bacterial resistance [46]. Concentrations of cefazolin measured in the saliva and sputum of the patients, similarly to our previous studies, indicated a very low penetration rate [30,31]. Thus, while circulating cefazolin levels appeared adequately high (corresponding to the highest MIC values measured in the oral bacterial cultures), the cefazolin levels in the saliva and bronchial secretions of the patients fell below 0.5 mg/L. In our two patient groups we could find no significant differences between the cefazolin levels of the serum, saliva or bronchial secretions. Furthermore, there were no differences in the type of bacteria isolated or the sensitivity of these bacteria to cefazolin between the control and the pneumonia groups.

It is noteworthy that the causative agents isolated from the sputum of patients, *Acinetobacter baumanii*, *Pseudomonas aeruginosa*, *Klebsiella pneumoniae *and *Escherichia coli*, had a relatively high cefazolin MIC (2–64 < mg/L). In contrast, the MIC of a number of species including *Staphylococcus aureus*, *Haemophilus influenzae *and most Streptococci fell around or below 0.5 mg/L indicating high sensitivity of these bacteria to cefazolin. It is possible therefore that cefazolin even at very low concentrations, affected the oral bacterial flora by suppressing the sensitive species. This in turn may have resulted in an overgrowth of the more resistant, Gram negative species, aspiration of which led to pneumonia in the susceptible patients. Our speculation is supported by the fact that skewing of the oral bacterial flora in the favor of the pathogenic Gram negative organisms can predispose to development of nosocomial pneumonia [59].

Even though pathogenic, Gram negative bacteria were isolated from the saliva of almost all patients we investigated; only those with severe periodontal disease developed pneumonia. Indeed it appears that the amount of bacteria aspirated plays a major pathogenic role in intubated patients [19]. Although we did not perform quantitative assessment of the bacteria isolated from the saliva of the patients, it is likely that more severe periodontal diseases are associated with a larger number of oral bacteria. Reduction of bacterial number by topical treatment therefore may be an attractive therapeutic choice for patients at high risk. For instance, use of chlorhexidine gluconate in the early post-intubation period was shown to mitigate or delay the development of ventilator-associated pneumonia [8,60-63]. Further, treatment of mechanically ventilated patients with a daily oral hygiene consisting of an 0.12% chlorhexidine gluconate wash reduced the risk for nosocomial pneumonia [63-65].

## Conclusion

Taken together, our study suggests that periodontal screening and a quantitative assessment of disease status is a useful method to identify patients at high risk of developing postoperative pneumonia. This measure is particularly important in elderly patients who commonly suffer from periodontal diseases. Based on our results a preoperative dental investigation and therapy for decreasing periodontal lesions is suggested in high risk patients in case of elective operation. Further studies to evaluate the role of bacterial load in the oral cavity and the effectiveness of topical treatment for prevention postoperative respiratory infections should be considered.

## Competing interests

The authors declare that they have no competing interests.

## Authors' contributions

KB carried out the dental investigations, participated in data acquisition, interpretation and manuscript preparation. AH participated in the statistical analysis and data interpretation. IM carried out the analysis of dental status. JS performed the microbiological tests. AG and AM carried out the measurements by capillary electrophoresis. IV made the specific sample collection and participated in data acquisition. JT carried out the patient selection and preoperative clinical investigations and helped to draft the manuscript. AK conceived of the study, and participated in its design, coordinated the investigations and has been involved in drafting the manuscript and giving final approval of the version to be published. All authors read and approved the final manuscript.

## Pre-publication history

The pre-publication history for this paper can be accessed here:

http://www.biomedcentral.com/1471-2334/9/104/prepub
